# Progress of the acyl-Coenzyme A thioester hydrolase family in cancer

**DOI:** 10.3389/fonc.2024.1374094

**Published:** 2024-03-18

**Authors:** Lu Bai, Pengjie Yang, Bater Han, Linghui Kong

**Affiliations:** ^1^ Inner Mongolia Medical University, Hohhot, China; ^2^ Department of Pathology, Peking University Cancer Hospital & Affiliated Cancer Hospital of Inner Mongolia Medical University, Hohhot, China; ^3^ Thoracic Surgery Department, Peking University Cancer Hospital & Affiliated Cancer Hospital of Inner Mongolia Medical University, Hohhot, China

**Keywords:** cancer, acyl-CoA thioester hydrolase, acyl-CoA hydrolase, Palmitoyl-CoA hydrolase, α/β-hydrolase fold enzyme superfamily, ‘hot dog’ fold superfamily

## Abstract

In recent years, the acyl-Coenzyme A thioester hydrolase family (ACOTs) has received wide attention as a key link in lipid metabolism. This family is a class of enzymes that catalyze the hydrolysis of fatty acyl-Coenzyme A, disrupting the thioester bond present within acyl-CoA ester molecules to produce free fatty acids (FFA) and the corresponding coenzyme A (CoA). Such enzymes play a very important role in lipid metabolism through maintaining appropriate levels of intracellular FFA and fatty acyl-CoA as well as CoA. It is broadly divided into two distinct subgroups, the type-I α/β-hydrolase fold enzyme superfamily and the type-II ‘hot dog’ fold superfamily. There are currently four human type-I genes and eight human type-II genes. Although the two subgroups catalyze the same reaction, they are not structurally similar, do not share the same sequence homology, and differ greatly in protein executive functions. This review summarizes the classification of the acyl-CoA thioester hydrolase family, an overview of the structural sequences, and advances in digestive, respiratory, and urinary systemic tumors. In order to explore potential specific drug targets and effective interventions, to provide new strategies for tumor prevention and treatment.

## 1 Cancer epidemiology studies

The increased burden of cancer is a global issue, closely related to factors such as an aging population and lifestyle changes (such as smoking and unhealthy eating habits), which partly reflects the level of social and economic development, which poses challenges to the development of effective cancer prevention and control and treatment strategies (1, 2). According to Global Cancer Statistics data in 2020, cancer incidence and mortality is rapidly increasing worldwide and may surpass cardiovascular disease as the leading cause of premature mortality in most countries (3). The latest data released by the China National Cancer Center shows that the incidence of cancer is lower compared with the United States and the UK, but the cancer mortality is higher. Meanwhile, the incidence of lung cancer, breast cancer, colorectal cancer and prostate cancer increase rapidly, and the incidence and burden of liver cancer, stomach cancer, esophageal cancer and cervical cancer are getting serious and heavier (4, 5). With the deeper understanding of the mechanisms of cancer occurrence and development, the diversity of cancer involves features such as genetic, cytology and tissue biology, pathology, and therapeutic response. In 2022, Douglas Hanahan (6) revised the characteristics of cancer to 14, giving us a better understanding of cancer to expand new therapeutic areas. Metabolic reprogramming is one of the hallmarks of malignant tumors, mainly showing enhanced glycolysis, active glutamine metabolism, and abnormal lipid metabolism (7). The acyl-CoA thioesterase family (ACOTs) is a group of enzymes that catalyze the hydrolysis of acyl-CoA esters into free fatty acids and coenzyme A, also known as “Acyl-CoA hydrolase”, “Acyl-CoA thioester hydrolase” or “Palmitoyl-CoA hydrolase”. This enzyme plays an important cellular role in fatty acid metabolism by regulating the cellular concentration of activated fatty acyl-CoA, so the ACOT gene is widely expressed in prokaryotes and eukaryotes (8, 9). Thus targeting lipid metabolic reprogramming plays an important role in supporting tumor progression and remodeling the tumor microenvironment, and targeting the ACOTs family in lipid metabolic pathways may expand new pathways for tumor prevention and treatment.

## 2 Overview of acyl-Coenzyme A thioesterase family (ACOTs) genes

### 2.1 Family members

Since the revision of its nomenclature by Hunt et al. in 2005 (10, 11), a total of 12 ACOT genes have been identified in the human genome, namely ACOT 1, ACOT 2, ACOT 4, ACOT 6-9, ACOT11-13, and two homologous protein—thioesterase superfamily members 4 (THEM 4) and 5 (THEM 5). Meanwhile, 15 were identified in murines, namely Acot 1-13, Them4 and Them 5.

They can be divided into two subsets, so-named Type-I and Type-II, based on their sequence and structural differences. According to their molecular weight, they can be further divided into type-I monomer and type-II polysome. The subcellular localization of these two types of Acots is diverse, type I Acots generally localized single sub-organelles, such as Acot 1 in cytoplasm, Acot 2 in mitochondria and Acot 4 in peroxidase. The subcellular localization of type II Acots has multiple localization phenomena, such as Acot 7, and Acot11-Acot 13 function in the cytoplasm and mitochondria (12). In addition, Them4 and Them 5 are located in the cytoplasm and mitochondria, respectively. ACOTs localized in peroxisome and mitochondria have signature substrate specificity, including saturated and unsaturated fatty acyl-CoA with different chain lengths and, at a lesser extent, other CoA esters. The main mechanism of action is through β-oxidation with chain shortening degradation of fatty acids as carboxylic acid excretion or transport to the mitochondria for further metabolism. For example, in **Table 1**, the aliases ACOT 2, ACOT 4 and ACOT 8 have PTE-2 (peroxisomal acyl-CoA thioesterase 2), which is the main acyl-coenzyme A thioesterase in peroxisome. In the regulation of β-oxidation of short chain acyl-CoA, β-oxidation of straight chain and branched chain fatty acids produces short acyl-CoA, which may be transferred to mitochondria (as carnitine ester) for further metabolism or excreted in urine. However, during β-oxidation of very long-chain acyl-CoA and long-chain acyl-CoA and dicarboxylic acids, acetyl-CoA is released and then transferred to carnitine by carnitine acetyltransferase, further metabolized or hydrolyzed in mitochondria to acetate and excreted into the cytoplasm (12). The study of Zhuravleva et al. (13) found that Acot 15 has a strong specificity for long-chain unsaturated fatty acid CoA esters, and is involved in the remodeling of cardiolipin and the development of fatty liver.

**Table 1:** Overview of the ACOTs family.

Subset	Subgroup	Murine	Full gene name	Chromosomal localization in house mouse	Chromosomal localization in Norway rat	Human	Chromosomal localization	Subunit localization	Aliases	Tissue expression	Disease
type-I monomer	α / β -hydrolase foldase superfamily	Acot1	Acyl-CoA thioesterase 1	12 D3	6q31	ACOT1	14q24.3	cytoplasm	ACH2,CTE-1,LACH2, Cte1,D12Ucla1	fat, liver, kidney	obesity; fatty liver, type 2 diabetes mellitus, gastric adenocarcinoma
		Acot2	Acyl-CoA thioesterase 2	12 D3	6q31	ACOT2	14q24.4	mitochondria	CTE-IA,CTE1A,MTE1, PTE2,PTE2A,ZAP128, MTE-I, Mte1	fat, liver, kidney	AML,colorectal cancer,lung cancer,breast cancer
		Acot3	Acyl-CoA thioesterase 3	12 D3	6q31						
		Acot4	Acyl-CoA thioesterase 4	12 D3	6q31	ACOT4	14q24.3	peroxisome	PTE-Ib,PTE1B,PTE2B	liver, kidney	pancreatic ductal carcinoma, gastric cancer, ischemic stroke
		Acot5	Acyl-CoA thioesterase 5	12 D3	6q31						
		Acot6	Acyl-CoA thioesterase 6	12 D3	6q31	ACOT6	14q24.3			the protein was not detected	
type-II polysomes	"hot dog" foldase superfamily	Acot7	Acyl-CoA thioesterase 7	4 E2	5q36	ACOT7	1p36.31	cytoplasm,mitochondria	ACH1,ACT,BACH, CTE-II, LACH, LACH1,hBACH	brain, kidney, small intestine	epilepsy (near middle temporal lobe), AML, gastric cancer, hepatocellular carcinoma
		Acot8	Acyl-CoA thioesterase 8	2 H3	3q42	ACOT8	20q13.12	peroxisome	HNAACTE,NAP1,PTE-1,PTE-2,PTE1, PTE2,hACTE-III,hTE	adrenal gland, brain	hepatocellular carcinoma,lung adenocarcinoma,renal cell carcinoma
		Acot9	Acyl-CoA thioesterase 9	X F3	Xq22	ACOT9	Xp22.11	mitochondria	ACATE2,MTACT48,MT-ACT48,CGI-16	heart,appendix,kidney,adipose tissue,brain	
		Acot10	Acyl-CoA thioesterase 10	15 A3							
		Acot11	Acyl-CoA thioesterase 11	4 C7	5q34	ACOT11	1p32.3	cytoplasm,mitochondria	BFIT,STARD14,THEA,THEM1	kidney, small intestine	chronic inflammation, obesity, diabetes mellitus, colorectal cancer, renal clear cell carcinoma,lung adenocarcinoma
		Acot12	Acyl-CoA thioesterase 12	13 C3	2q12	ACOT12	5q14.1	cytoplasm,mitochondria	CACH-1,Cach, STARD15,THEAL	liver, kidney	hepatocellular carcinoma,intrahepatic cholangiocarcinoma,non-alcoholic fatty liver disease, psoriasis,glioma
		Acot13	Acyl-CoA thioesterase 13	13 A3	17p11	ACOT13	6p22.3	cytoplasm,mitochondria	HT012,PNAS-27,THEM2	liver,kidney,intestine,heart,brown fat	lung adenocarcinoma, hereditary polycystic kidney disease, ovarian serous cystic adenocarcinoma
		Them4	thioesterase superfamily member 4	3 F2	2q34	THEM4	1q21.3	cytoplasm	CTMP	kidney, testis	glioblastoma, TNBC, lung cancer, SCCHN
		Them5	thioesterase superfamily member 5	3 F2	2q34	THEM5	1q21.3	mitochondria	Acot15	skin, brain	fatty liver disease, AIDS

### 2.2 The structural sequence

The subgroup of monomers type-I are members of the α/β-hydrolase fold enzyme superfamily. Human type-I ACOTs contain ACOT 1, ACOT 2, ACOT 4, and ACOT 6, and all four genes form an 80-base gene cluster on chromosome 14q24.3 (8, 14). Murine type-I Acots contain Acot 1-6, and its house mouse gene clusters all show the formation of base clusters within 120 kb on chromosome 12 D3 (10, 15), A highly conserved amino acid sequence was also found on chromosome 6q31 in the Norway rat. Thus Brocker C et al. (8) speculated that type-I proteins may arise due to gene duplication. Type-I proteins have a high degree of sequence homology, with 98% amino acid sequence identity between ACOT 1 and ACOT 2; ACOT 4 shares 70% identity with ACOT 1 and ACOT 2, while ACOT 6 is the smallest, but still has 54%-57% sequence identity with other members. Interestingly, none of these proteins have any significant sequence homology with type-II proteins. All additional type-I proteins contain an esterase-lipase superfamily domain at the C terminus, also known as the α/β -hydrolase fold, that catalyzes all the required residues. In addition to ACOT 6, three other type-I proteins contain an acyl-Coenzyme A thioester hydrolase domain at the N terminus, which may have some distant structural homolog with other proteins. To date, no full-length enzyme or recombinant protein expression has been detected by ACOT 6 in the subgroup of human monomer type-I.

The subgroup of type-II polysomes belongs to the ‘hot dog’ fold superfamily. There are eight human type II ACOTs, namely ACOT 7-9, ACOT11-13, THEM 4, and THEM 5. Mouse type II ACOTs contain Acot 7-13, Them 4, and Them 5. This subgroup has significantly lower sequence homology than the type-I proteins. According to the results of the nucleic acid sequence database BLAST, all type-II proteins have less than 25% identity, and many proteins do not have any significant sequence similarity. However, in the structure of mammalian systems, all type-II proteins have a common structural feature, namely, two consecutive ‘hot dog’ folded domains at the N terminus (except only one ACOT13) and one C-terminal START (StAR associated lipid transfer) domain (16). According to the experimental results of Pidugu LS et al. (17–19), the outer seven anti-parallel β fold “bread” wraps a hydrophobic curved α spiral “sausage”, and a layer containing of circles on the helix acts as a cover loop. The active site involved in the reaction is located at the dimer interface between the two ‘hot dog’ fold domains, consisting of the catalytic triad linked to the α helix and the β chain—serine, histidine and aspartate. Interestingly, several proteins with this ‘hot dog’ fold domain are all involved in fatty acid elongation, thioester hydrolysis, and transcriptional regulation in fatty acid metabolism.

Furthermore, the StART (steroidogenic acute regulatory protein-related lipid transfer) domain is a protein module of about 210 residues and can bind to various lipids to mediate intracellular functions, including intracellular lipid transport, lipid metabolism and cell signals (9, 20). The structure of the StART domain in Kirkby (9) is inferred as: an antiparallel β fold consisting of 9 strands surrounds five α helices, and a tunnel consisting of one β layer and three α layer helices surrounds a single pentagonol molecule. The key function of this domain is to bind lipids.

Although the two types catalyze the same reaction, the type-I and type-II enzymes have no structural or sequence similarity, and the translated proteins have different domains, suggesting that the type-I and type-II enzymes are not homologous.

## 3 Progress of ACOTs in cancer

### 3.1 ACOTs and digestive system tumors

Currently, ACOT 1, ACOT 4 and ACOT 7 in this family are extensively studied in stomach cancer (**Table 2**). Wang F et al. (21) by 280 cases of gastric adenocarcinoma tissue microarray expression and comprehensive analysis found ACOT 1 expression up-regulated in gastric adenocarcinoma tissue, and closely related to the clinical pathological parameters and poor prognosis of gastric adenocarcinoma, but the specific mechanism is not clear. Wang F et al. (21) speculated that ACOT 7 may induce GC progression by increasing the expression of the potential tumor-promoting protein GLI 3 (Gli family zinc finger structure 3). LiQ et al. (22) later confirmed that ACOT 4 was also up-regulated and had a poor prognosis in gastric adenocarcinoma tissues by the tissue microarray method.

**Table 2:** Progression of human ACOTs members in tumors.

		ACOT1	ACOT2	ACOT4	ACOT7	ACOT8	ACOT11	ACOT12	ACOT13	THEM4
digestive system tumors	stomach cancer	√		√	√					
	colorectal cancer		√				√			√
	liver cancer				√	√		√		
	pancreatic cancer			√						
respiratory system tumors	lung cancer		√		√	√	√		√	√
urological system tumors	renal cancer					√	√			
other systemic tumors	glioma							√		
	glioblastoma									√
	AML		√		√					
	breast cancer		√							√
	ovarian serous cystadenocarcinoma								√	
	SCCHN									√

In addition to its participation in the poor prognosis of gastric cancer, ACOT 4 also plays an important role in the development of pancreatic ductal cancer and poor survival. NiC et al. (23) suggested that the cumulative excess of ACOT 4 promotes the metabolism of tumor cells and induces the development of pancreatic cancer by producing excessive coenzyme A. Feng H et al. (24) also detected high expression of ACOT 7 in gastric cancer tissues and cell lines. Its deletion would increase apoptosis of gastric cancer cells, effect cell cycle changes, and also reduce the growth and tumorigenicity of gastric cancer cells.

In liver cancer, Hung YH et al. (25) found that ACOT 8 was highly expressed in peroxisome of hepatoma tissues, and its protein expression and overall thioesterase activity after the knockout of ACOT 8 decreased and inhibited the cell proliferation and tumorigenicity of hepatocellular carcinoma. Xie X et al. (26) found that the indirect mechanism by which ACOT 7 over-expression promotes the progression of HCC was that over-expression of ACOT 7 increased the production of monounsaturated fatty acid oleic acid (C18:1). For ACOT12, the research focuses more on the role of liver cancer formation and metastasis. Recently, it has been shown that the expression of ACOT12 (27–29) is significantly down-regulated in hepatocellular carcinoma and hepatic cholangiocarcinoma tissues, and the tumor metastasis is suppressed. The mechanism may be that ACOT12 regulates acetyl-CoA levels and histone acetylation in cancer cells, silencing ACOT12 can induce the expression of oncogene TWIST2 or Snail superfamily Slug, and promote epithelial-mesenchymal transition (EMT) to promote liver cancer metastasis. In addition, Acot12 can inhibit hepato-carcinogenesis by limiting the biosynthesis of glycerolipid (including lysophosphatidic acid LPA), pro or associated with reduced Hippo signaling and increased transcriptional activity mediated by the effector molecule YAP.

In colorectal cancer, Zhang S et al. (30) recently identified THEM 4 in the acyl-CoA thioesterase family in studying the effects of exosomal miR-183-5p enriched in M2 polarized tumor-associated macrophages (TAM) on colon cancer cells. Over-expression of THEM 4 alleviates miR-183-mediated oncogenesis by 5p and inactivates Akt and NF- κ B pathways in colon cancer cells, thereby inhibiting their proliferation, migration and invasion. Asghari Alashti F et al. (31) used bioinformatics analysis of gene expression characteristics of colorectal cancer patients and found that ACOT 11 showed low expression in cancer tissues compared with adenoma and normal tissues, so they speculated that ACOT 11 could be used as a biomarker for early detection and treatment of colorectal cancer. However, this conclusion is only drawn from the data analysis, and the specific mechanism of the biological function of ACOT 11 in colorectal cancer still needs to be confirmed by many experimental studies and clinical studies. Jung EJ et al. identified ACOT 2 as an oxaliplatin receptor-related protein (32), and speculated that ACOT 2 is involved in drug resistance in metastatic colorectal cancer.

### 3.2 ACOTs and respiratory system tumors

The acyl-Coenzyme A thioesterase family has also had many genes studied in lung cancer, such as ACOT 2, ACOT 7, ACOT 8, ACOT11, ACOT13, and THEM 4.

In addition to the association with poor prognosis of ACOT 7 in gastric cancer, Wang T et al. (33) found that high ACOT 7 expression was also associated with poor prognosis in patients with non-small cell lung cancer. Loss-of-function and gain-of-function experiments showed that ACOT 7 promotes the growth and proliferation of NSCLC and inhibits apoptosis and ferroptosis in NSCLC, but has no effect on cell cycle progression. ACOT 7 over-expression also enhances fatty acid synthesis and suppresses lipid peroxidation, plays a necessary role in the oncogenesis of ARNTL2/ACOT 7 axis in non-small cell lung cancer. And could reverse inhibition or promote cellular triglyceride production and subsequent cell proliferation by ARNTL2 downregulation or overexpression of ACOT 7. Jung SH et al. (34) speculated that reduced ACOT 7 activity may be involved in preventing the development of human breast and lung cancers through regulation of cell cycle progression. Further demonstrated that ACOT 7 plays a critical role in cell cycle progression and may become an important target of chemoradiotherapy in lung cancer. In addition to ACOT 7, ACOT 2 has also been implicated in NSCLC, and Guo et al. (35) demonstrated based on the human metabolic network that ACOT 2 can participate as a potential molecular target of NSCLC in the metabolic pathways of aromatic amino acids and long-chain fatty acids.

ACOT 8 (36) is a specific protein associated with lymph node metastasis and prognosis in lung adenocarcinoma, and its high expression was significantly associated with an increased risk of cancer-related death, independent of clinical factors.

Liang C et al. (37, 38) By exploring the regulatory mechanism of ACOT 11 in lung cancer, they found that ACOT 11 is located in cytoplasm and was highly expressed in lung squamous cell carcinoma and different subtypes of adenocarcinomas (including papillary adenocarcinoma and mucinous adenocarcinoma). Knockdown of ACOT 11 suppressed the conversion of G1 to S phase in the lung cancer cell cycle and clonogenic formation of lung cancer cells; it could also inhibit the tumor growth and the migration and invasion of lung cancer cells by blocking the epithelial-mesenchymal transformation. Subsequently, Liang C et al. (37) speculated through immunoprecipitation-mass spectrometry analysis that the underlying molecular mechanism of ACOT 11 may be combined with CSE 1 L (human cell apoptosis susceptibility gene, also known as XPO 2), which regulates the proliferation, migration and invasion of lung cancer through various signaling pathways. Hung JY et al. (38) yielded consistent results from experimental studies of the proliferative effects of ACOT11 and ACOT13 in lung adenocarcinoma. Huang SK et al. (39, 40) found that THEM 4 (carboxy-terminal regulatory protein, CTMP) as a mitochondrial protein has a certain role in lung cancer, which can prevent the interaction between cytochrome c and APAF-1 (apoptosis enzyme activator) by inhibiting heat shock protein 27, so as to promote mitochondria-mediated cell apoptosis, and then inhibit the progression of lung cancer. Its over-expression can cause mitochondrial membrane depolarization and increased caspase-3 increase, and promote cell apoptosis by delaying the protein kinase (PKB/Akt) phosphorylation after cell death induction.

### 3.3 ACOTs and urological system tumors

ACOT 8 not only functions as an independent predictor of lymph node metastasis and lung adenocarcinoma survival in lung adenocarcinoma lymphatic metastasis and hepatocellular carcinoma development, but also plays a crucial role in clear cell renal cell carcinoma. XuCL et al. (41) explored the potential value of ACOTs in clear cell RCC (renal-cell carcinoma) patients through bioinformatics analysis, and found that the expression of ACOT 8 and ACOT11 in clear cell RCC was significantly down-regulated. The difference between the two factors was that the differential expression of ACOT11 was of potential value in the diagnosis of clear cell RCC; while the transcript expression of ACOT 8 was increasing with the progression of tumor, and only the expression of ACOT 8 was closely correlated with histological grade and poor prognosis. The multivariate Cox regression analysis found ACOT 8 as the only independent prognostic marker in ACOTs with predictive value. XuCL et al. hypothesized that ACOT8 might promote oxidative phosphorylation and inhibit ferroptosis by regulating mitochondrial electron transport in clear cell renal cell carcinoma to affect the occurrence and progression of renal cell carcinoma (41). Since the results of the above views are from big data, further clinical trial studies are needed to confirm this speculation.

### 3.4 ACOTs with other systemic tumors

In tumors of the nervous system, gliomas are frequently closely associated with ACOT12, and recently it has been shown that exosomes released from glioma stem-like cells (GSC) can reduce the expression of ACOT12 and promote epithelial mesenchymal transition, thus promoting the invasive growth of glioma. As mentioned above, ACOT12 can promote metastasis by regulating the key transcription factor TWIST2 in EMT, and in the study by Bao Z et al. (42), ACOT12 can also act as a tumor suppressor gene in gliomas by modulating the oncogenes TWIST2 and EMT in gliomas. Wang J et al. (43–45) found that THEM 4 acts as an endogenous inhibitor of protein kinase B (PKB/Akt) in glioblastoma, which preferentially binds phosphorylated Akt and blocks downstream signaling by eliminating Akt activity and phosphorylation. When the function or expression of THEM 4 is absent, the inhibition of Akt is abolished, thereby promoting tumorigenesis. Considering this function of THEM 4 (CTMP), Lin CH et al. (46) showed that THEM 4 promotes the metastasis of triple-negative breast cancer (TNBC) through the Akt activation-dependent pathway. Chen YC et al. (47) found in their experiments that THEM 4 (CTMP) was highly expressed in the trastuzumab unresponsive group and was positively correlated with Akt activity, indicating that CTMP promotes Akt activation leading to resistance to trastuzumab in HER 2-positive breast cancer patients. Therefore, they speculated that the expression of CTMP in clinical drug resistance could be used as a prognostic indicator for HER 2-positive breast cancer. In addition, CTMP can also promote tumor cell proliferation and EMT in (48) by regulating Akt phosphorylation, up-regulating Snail and down-regulating E - cadherin, and then promoting tumorigenesis and metastasis. It is worth mentioning that CTMP inhibition can restore sensitivity to cisplatin chemotherapy, and targeting CTMP may provide a new perspective for the treatment of SCCHN (squamous cell carcinoma of the head and neck).

In acute myeloid leukemia (AML) of the blood system (49, 50), ACOT 2 and ACOT 7 plays an important role in lipid metabolism such as fatty acid elongation and the biosynthesis of unsaturated fatty acids. Among them, ACOT 2 was up-regulated in AML cell lines, and its high expression predicted decreased overall survival and abnormal lipid metabolism during disease progression in AML. Similarly, high expression of ACOT 7 is also closely associated with poor prognosis in AML. In addition, ACOT 2 expression was also increased in mitochondria of breast cancer cell lines and induced arachidonic acid (AA) release from arachidoidyl-coenzyme A (AA-COA) and is associated with the expression of the arachidonylase ACSL 4 (51).

In reproductive system tumors, some studies have analyzed the clinicopathological parameters and the relationship of prognosis between ACOT13 and ovarian serous cystadenocarcinoma (52) by bioinformatics,and found that ACOT13 had low expression in normal ovarian tissue, but high expression in ovarian serous cystadenocarcinoma tissue, and correlated with the tumor stage. This differential expression has a large impact on tumor formation and immune invasion. However, the relevant data is limited, and the carcinogenic mechanism and clinical application value of ACOT13 in ovarian cancer need to be further studied.

## 4 Outlook

In recent years, many major advances in cancer biology, epigenetics, and bioinformatics machine learning have been made in the functional and structural characterization of the mammalian acyl-CoA thioesterase family. Elucidation of its functional roles has revealed associations with lipid biosynthesis, allosteric regulation of enzymes, regulation of ion channel opening, signaling, budding and fusion of the inner cellular membrane, and regulation of gene transcription through nuclear receptors. While recent studies have greatly expanded the current understanding of these proteins and their physiological importance, there are still some members whose functions are relatively unexplored and warrant further investigation.

## Author contributions

LB: Writing – original draft, Conceptualization, Methodology. PY: Writing – review & editing, Conceptualization, Methodology, Funding acquisition. LK: Writing – review & editing, Conceptualization, Methodology, Project administration, Funding acquisition. BH: Writing – review & editing, Formal analysis, Funding acquisition.
